# Blockade of PD-1/PD-L1 Promotes Adoptive T-Cell Immunotherapy in a Tolerogenic Environment

**DOI:** 10.1371/journal.pone.0119483

**Published:** 2015-03-05

**Authors:** Stephen J. P. Blake, Alan L. H. Ching, Tony J. Kenna, Ryan Galea, Justin Large, Hideo Yagita, Raymond J. Steptoe

**Affiliations:** 1 UQ Diamantina Institute, University of Queensland, Brisbane, Australia; 2 Department of Immunology, Juntendo University School of Medicine, Tokyo, Japan; Maisonneuve-Rosemont Hospital, CANADA

## Abstract

Adoptive cellular immunotherapy using in vitro expanded CD8^+^ T cells shows promise for tumour immunotherapy but is limited by eventual loss of function of the transferred T cells through factors that likely include inactivation by tolerogenic dendritic cells (DC). The co-inhibitory receptor programmed death-1 (PD-1), in addition to controlling T-cell responsiveness at effector sites in malignancies and chronic viral diseases is an important modulator of dendritic cell-induced tolerance in naive T cell populations. The most potent therapeutic capacity amongst CD8^+^ T cells appears to lie within Tcm or Tcm-like cells but memory T cells express elevated levels of PD-1. Based on established trafficking patterns for Tcm it is likely Tcm-like cells interact with lymphoid-tissue DC that present tumour-derived antigens and may be inherently tolerogenic to develop therapeutic effector function. As little is understood of the effect of PD-1/PD-L1 blockade on Tcm-like CD8^+^ T cells, particularly in relation to inactivation by DC, we explored the effects of PD-1/PD-L1 blockade in a mouse model where resting DC tolerise effector and memory CD8^+^ T cells. Blockade of PD-1/PD-L1 promoted effector differentiation of adoptively-transferred Tcm-phenotype cells interacting with tolerising DC. In tumour-bearing mice with tolerising DC, effector activity was increased in both lymphoid tissues and the tumour-site and anti-tumour activity was promoted. Our findings suggest PD-1/PD-L1 blockade may be a useful adjunct for adoptive immunotherapy by promoting effector differentiation in the host of transferred Tcm-like cells.

## Introduction

One approach to overcoming loss of effective anti-tumour immunity in tumour-bearing patients is adoptive cellular immunotherapy. T cells with tumour-antigen specificity are isolated from the patient or engineered ex-vivo and expanded prior to reinfusion. Mouse tumour models have suggested that central memory (Tcm) phenotype CD8^+^ T cells or T memory stem cells (Tscm) that possess potent expansion potential, but little inherent cytotoxic activity [1], are most effective for immunotherapy in this setting [2,3]. In humans, Tcm-derived cells may exhibit superior engraftment properties, although this is yet to be fully defined [4,5]. Adoptive immunotherapy has shown promise in the clinic [6] but has been limited by the failure to persist and loss of function of the transferred T cells [7,8]. In tumour-bearing individuals, inhibition of T-cell effector function at the tumour site is not the only impediment to immune-mediated tumour clearance. Soluble factors released from the tumour environment can systemically impair DC maturation and function. In addition, tumour-derived exosomes [9] and DC migrating from tumours [10] may act to tolerise T cells distant from the tumour site leading to a loss of functional tumour-specific T cells. Previously we have shown that effector and memory T cells are particularly susceptible to inactivation by steady-state DC presenting cognate antigen [11,12] much as they might be in a tumour-bearing host. Therefore, DC presenting tumour antigens could act to inhibit the function of adoptively-transferred CTL or CD8^+^ Tcm-like cells.

In the ‘classical’ model of T-cell stimulation, antigen and co-stimulation combine to control the outcome of T-cell activation viz-a-viz immunity or tolerance. However, in addition to ‘activating’ costimulatory molecules such as CD28, ‘co-inhibitory’ molecules also act to limit T-cell function. A growing number of co-inhibitory molecules belonging to the CD28 and other gene families have been described [13]. These molecules are of significant therapeutic interest as their manipulation could enhance or limit T-cell responses. One pathway that has been a focus of interest is the CD28 family member programmed death-1 (PD-1)^3^ and its ligands programmed death-ligand 1 (PD-L1; B7–H1) and programmed death-ligand 2 (PD-L2; B7-DC) [14].

PD-1 is expressed at low levels on naive T cells, but is upregulated upon T-cell activation [15]. In situations of chronic antigen stimulation, PD-1 remains elevated on T cells and is a marker of chronic antigen-exposure [16]. Signalling through PD-1 limits T-cell function both during priming [17,18] and at effector sites [19] where the role of PD-1 may be greater. The two PD-1 ligands differ in expression patterns. PD-L1 is widely and constitutively expressed on a range of cell types whereas PD-L2 expression is restricted primarily to activated dendritic cells (DC) and myeloid cells [15]. PD-1/PD-L1 signalling is exploited by viruses and tumours to evade immune destruction. In a wide range of chronic viral infections PD-L1 is expressed or upregulated on infected tissues and PD-L1 is expressed by a range of tumour types and has been associated with a poor prognosis [13]. In conjunction with prolonged PD-1 expression associated with chronic antigen stimulation, PD-1/PD-L1 interactions contribute to T-cell ‘exhaustion’ [16]. Under these conditions, PD-1/PD-L1 blockade can provide some rescue of effector T cell function [16] and recent clinical trials of PD-1 and PD-L1 blockade in cancer have shown very promising outcomes [20,21]. It is generally considered, that for enhancing anti-tumour immunity, PD-1/PD-L1 blockade acts to promote T-cell effector activity at the tumour site [22,23]. PD-1 ligation also functions in a similar way to limit effector function in sites subject to autoimmune effector function [24]. But in addition PD-1 plays an important role in modulating the outcome of the initial activation of naive T cells and together with CTLA4 has been reported to modulate tolerance induction in naive T cells [25]. Little is known of how PD-1/PD-L1 might regulate memory CD8^+^ T-cell responses, with studies typically focussed on dysfunctional CD8^+^ ‘helpless’ memory populations that arise in chronic infection. PD-1 expression is typically higher on memory CD8^+^ T cells than naive counterparts and as adoptively transferred tumour-specific Tcm-phenotype CD8^+^ T cells, in keeping with established trafficking patterns for Tcm [26] and Tcm-like cells [27], likely first encounter DC presenting tumour antigens in lymphoid tissues, where differentiation to effectors may occur we set out to examine this. Furthermore, while a role for PD-1 in modulating tolerance induction in naive T cells has been established for naive CD8^+^ T cells this is yet to be examined for memory or effector CD8^+^ T cells.

Here we set-out to explore this and the impact of PD-1/PD-L1 blockade in a model of adoptive tumour therapy where tolerising DC are present. We demonstrate that blockade of PD-1/PD-L1 impaired inactivation of CD8^+^ Tcm-phenotype cells adoptively transferred into a strongly tolerogenic environment, promoted effector differentiation and positively impacted the effectiveness of Tcm-phenotype CD8^+^-mediated adoptive tumour immunotherapy. These data indicate blockade of PD-1/PD-L1 interactions may be useful in adoptive cellular immunotherapy using expanded tumour-specific T cells.

## Materials and Methods

### Mice

Male or female (C57BL/6J or C57BL/6.SJL*ptprca*) were from the Animal Resources Centre (Perth, Australia) or bred and maintained at the Biological Research Facility (Woolloongabba, Australia) under SPF conditions. CD45.1^+^ OT-I mice were generated by breeding OT-I mice [28] with C57BL/6.SJL*ptprca* mice. 11c.OVA mice have been described [29]. Mice were allocated to experiments randomly and typically used at 6–12 weeks of age.

### Ethics Statement

All experiments were approved by the University of Queensland Animal Ethics Committee (Projects 047/10, 076/13).

### Cell preparation and transfer

For naïve T cell transfers, pooled brachial, axillary, inguinal and mesenteric LN cells from CD45.1^+^ OT-I mice were injected i.v. (5x10^6^, corresponding to 2x10^6^ CD8^+^ OT-I T cells). For transfer of CD8^+^ Tcm-phenotype cells and adoptive immunotherapy we used our modification of procedures described previously [27,30]. Pooled OT-I LN cells were cultured as described [11] in complete RPMI 1640 containing OVA_257–264_ (0.1 μg/ml) and rhIL-2 (10 ng/ml) for three days, harvested, washed and recultured in the presence of rmIL-15 for a further 2 days. This culture procedure generated a population of post-activated CD8^+^ OT-I T cells comprising predominantly Tcm-phenotype cells (typically ≥90%, (94 ± 7% mean ± SD, n = 17) CD44^hi^CD62L^hi^) with a minority of CD44^hi^CD62L^lo^ Tem phenotype cells (S1 Fig.). These are referred to here as Tcm-phenotype cells. After harvesting and washing, 2x10^6^ cells (>95% CD8^+^/Vα2^+^) were transferred i.v.

### Antibodies, in vivo and in vitro analyses

Flow cytometry mAb were from BioLegend (San Diego, CA, USA), BD (San Jose, CA, USA) or eBioscience (San Diego, CA, USA). αPD-1 (RMP1–14) [31], αPD-L1 (10F-9G2) [32] and isotype control (LTF-2 or MAC4) were purchased from BioXCell (Lebanon, NH, USA) or purified from hybridoma supernatants in house and injected i.p. (200 μg every 3 days as required) as reported [16,33] and the blocking activity in vivo verified. B16.mOVA was generated by transfection of B16-F0 cells (B16-F0 purchased from ATCC; Cat CRL-6322) with pcDNA3.1 encoding a human transferrin receptor/ovalbumin fusion protein [29] under the CMV promoter. Stable transfectants were selected with zeocin (Sigma-Aldrich, St Louis, MO, USA) and genotype confirmed by PCR. B16.mOVA and parental B16F0 were cultured as described by the supplier (ATCC) but with complete RPMI/10% FCS, harvested, washed and unless stated otherwise 1x10^5^ injected s.c. Tumour size was determined in a blinded fashion and area calculated by multiplying diameters of 2 perpendicular axes. Mice were euthanized when tumours exceeded 1cm x 1cm. Where indicated mice were immunized s.c. at the tail-base with OVA/QuilA (100 μg OVA [Grade V, Sigma], 20 μg QuilA [Soperfos Biosector DK-Vedback, Denmark]). Tumour single cell suspensions were prepared by gently disrupting tumours before digestion (complete RPMI/ 1 mg/ml collagenase D (Roche) /100 μg/ml DNAse (Roche) 60 min, 37°C with gentle mechanical disruption). Tumour digests were filtered (70 μm cell strainer, BD) and washed twice with cold PBS 2.5% FCS. Lymphoid tissues were prepared, stained and analyzed as described previously [29]. In vivo CTL assays were performed as described [29]. Briefly, syngeneic spleen cells pulsed or not with OVA_257–264_ (0.02 ug/ml) for 1 h at 37°C, washed, and labeled with 5 uM CFSE or 0.5 uM CFSE, respectively; 10^7^ cells from each population were then injected i.v. Three hours later, spleens were collected and single cell suspensions analyzed by flow cytometry with propidium iodide dead cell exclusion. CTL activity was determined using: percentage of killing = [1–test (counts^unpulsed^/ counts^pulsed^) ÷_ control (counts^unpulsed^/counts^pulsed^)] x 100.

### Statistical analyses

Students *t*-test was used to compare means and one way ANOVA followed by Newman-Keuls post test (GraphPad Software, San Diego, CA, USA) for multiple pairwise comparisons. Comparison of average tumour size was made at a time point where 90% of untreated control mice had reached maximum ethically-approved tumour size (1cm^2^).

## Results

### Blockade of PD-1/PD-L1 impairs DC-mediated tolerance in naïve CD8^+^ T cells

**Fig 1:**
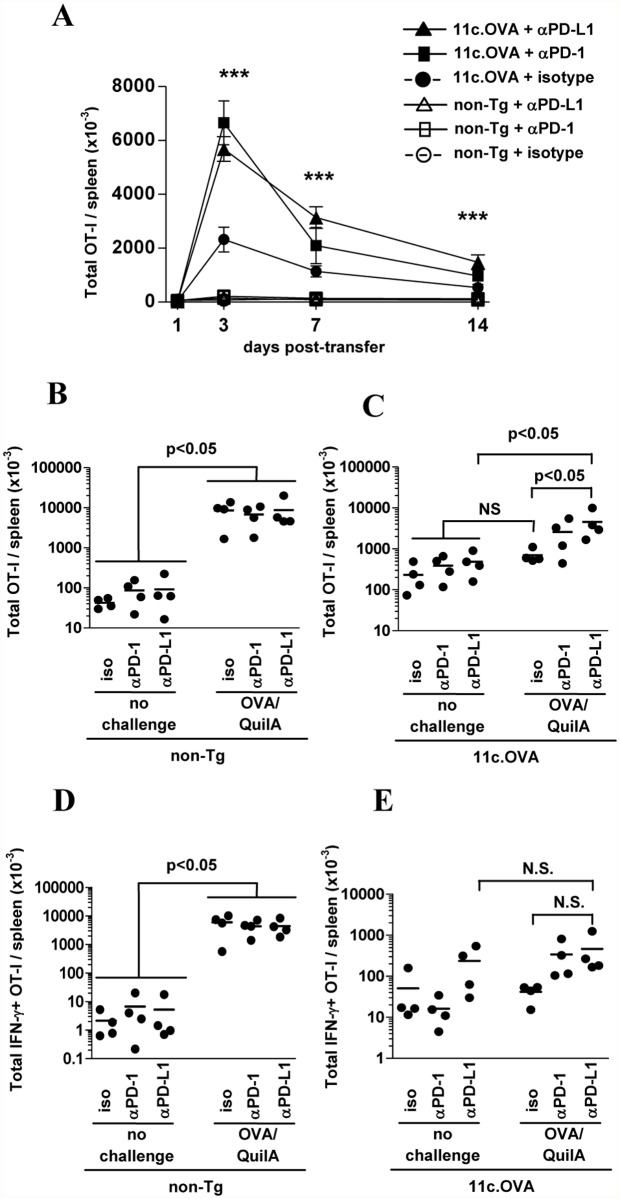
Blockade of PD-1/PD-L1 impairs induction and partially reverses tolerance in naïve CD8^+^ T cells. A) CD45.1^+^ OT-I T cells were transferred to 11c.OVA or C57BL/6 (non-Tg) mice treated with αPD-1, αPD-L1 or isotype control mAb at transfer and every subsequent three days. At the indicated time points after transfer spleens were collected and OT-I T cell number determined by a flow-cytometric counting assay. B-E): CD45.1^+^ OT-I T cells were transferred to C57BL/6 (non-Tg) or 11c.OVA mice and 21 days later mice were treated with αPD-1, αPD-L1 or isotype control mAb every three days. Six days after commencement of mAb treatment some mice were challenged with OVA/QuilA immunization and a further five days later mice were euthanized and tissues collected for analysis using a flow-cytometric counting assay and intracellular cytokine staining. (A) ***: for 11c.OVA recipients, αPD-L1 significantly greater than isotype control at day 3, 7, 14 (p<0.001), αPD-L1 significantly greater than αPD-1 at d7 (p<0.001) and d14 (p<0.05), αPD-1 greater than isotype at day 3 (p<0.001) and day 7, 14 (p<0.05). Data comprise: (A) four to seven mice for each time point (mean ± SEM) pooled from two or more experiments with 2–3 mice per group, (B-E) data pooled from 2 or 3 individual experiments with 1–2 mice per group (n = 4 for all groups) with values for individual mice shown.

As PD-1 ligation influences tolerance induction in naive T cells we first tested whether, in our hands, blockade of PD-1 or PD-L1 modulated the responses of naïve CD8^+^ T cells to tolerogenic steady-state DC using 11c.OVA mice where DC express OVA and present OVA-derived peptides [29]. After transfer to 11c.OVA mice, OVA-specific CD8^+^ TCR transgenic (OT-I) T cells undergo substantial, but abortive proliferation (expansion phase) followed by deletion (contraction phase) and a residual population of unresponsive ‘tolerant’ OT-I T cells remains (tolerance phase). Administration of blocking αPD-1 or αPD-L1 significantly increased the magnitude of OT-I accumulation 3 days after transfer and prolonged persistence of the expanded OT-I population in 11c.OVA recipients (Fig. 1A). This indicated that the antibodies were working effectively and by reproducing the findings of others that PD-1 or PD-L1 blockade modulates early activation events in CD8^+^ T cells [18], validated the experimental system.

### Blockade of PD-1/PD-L1 partially reverses DC-mediated ‘exhaustion’ of transferred naïve CD8^+^ T cells

Blockade of PD-1 signalling can partially restore effector function in CD8^+^ T cells ‘exhausted’ by chronic antigen presentation and high local expression of PD-1 ligands [16]. To determine whether blockade of PD-1 or PD-L1 once tolerance was established would reverse DC-induced CD8^+^ T-cell inactivation, OT-I were transferred to 11c.OVA mice and, 21 days later, at a time point, when OT-I T cells are rendered unresponsive [29], αPD-1 or αPD-L1 was administered. In non-transgenic recipients, challenge with OVA/QuilA resulted in substantial (>80-fold) expansion of OT-I cells not altered by αPD-1 or αPD-L1 treatment (Fig. 1B). Isotype control treatment failed to restore OT-I expansion (Fig. 1C) whereas αPD-L1 restored a modest but statistically-significant (7-fold) expansion of OT-I T cells after OVA/QuilA challenge (Fig. 1C). αPD-1 administration mediated an intermediate, non-significant effect. To determine whether this corresponded to increased effector function, production of IFN-γ was measured. In non-transgenic recipients, OVA/QuilA challenge induced a substantial increase (>1000-fold) in IFN-γ-producing OT-I T cells not altered by αPD-1 or αPD-L1 (Fig. 1D). Whilst there was a trend toward an increase in the total number of OT-I producing IFN-γ after administration of αPD-1 or αPD-L1, this failed to reach statistical significance, most likely due to the low number of mice sampled (Fig. 1E).

Overall these data indicate that in the 11c.OVA model, blockade of PD-1 or PD-L1 promotes OT-I T-cell expansion in response to chronic ‘tolerogenic’ DC-presented antigen and αPD-L1 partially restores proliferative capacity in ‘inactivated” OT-I T cells consistent with the findings in other experimental systems [16]

### Anti-PD-L1 promotes effector function of CD8^+^ Tcm-phenotype cells transferred to a tolerogenic environment

The influence of PD-1/PD-L1 on T cell function has principally been investigated using naive T cells. However, differentiated CTL or CD8^+^ Tcm-phenotype cells can be used for adoptive immunotherapy and these are susceptible to the influence of tolerogenic APC [11]. As Tcm-phenotype cells show the greatest efficacy for tumour clearance [2], we investigated whether PD-1/PD-L1 could influence their function in a tolerogenic environment. Expression of PD-1 increased on OT-I T cells activated and differentiated into effector cells in the presence of OVA_257–264_ and IL-2 (day 0–3 of culture, S1 Fig.). When OVA_257–264_ and IL-2 was washed off and replaced with IL-15 to generate Tcm-phenotype cells, PD-1 expression was reduced somewhat but remained present at an elevated level compared to naive OT-I. PD-L1 was upregulated by activation, but returned to resting levels as Tcm-phenotype cells differentiation occurred and, as expected, PD-L2 was not expressed.

**Fig 2:**
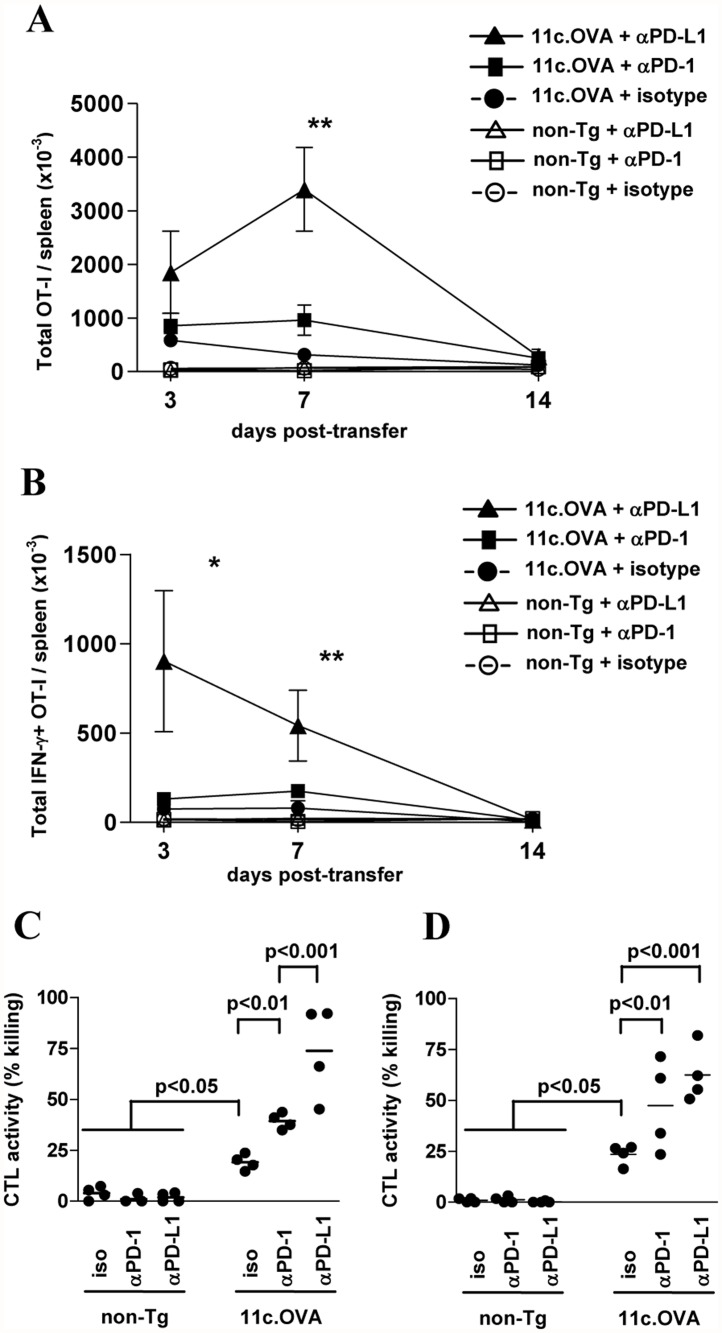
Blockade of PD-1/PD-L1 impairs tolerance in memory CD8^+^ T cells. A, B) In vitro generated CD45.1^+^ OT-I memory T cells were transferred to 11c.OVA or C57BL/6 (non-Tg) mice treated with αPD-1, αPD-L1 or isotype control mAb at transfer and every subsequent three days. At the indicated time points spleens were collected and OT-I T cell number (A) and total IFN-γ-producing OT-I number (B) determined by a flow-cytometric counting assay and intracellular cytokine staining. C, D) In vitro generated CD45.1^+^ OT-I Tcm-phenotype cells were transferred to 11c.OVA or C57BL/6 (non-Tg) mice treated with αPD-1, αPD-L1 or isotype control mAb at transfer and every subsequent 3 days and seven (C) or 14 (D) days later CTL activity determined in vivo. Data comprise: (A) 4–6 mice for each time point (mean ± SEM) pooled from two or more experiments of 2–3 mice per group, ** 11c.OVA + αPD-L1 significantly greater than 11c.OVA + isotype and 11c.OVA + αPD-1 at day 7 (p<0.01), (B) 4–6 mice for each time point (mean ± SEM) pooled from more than two experiments of 2 mice per group, * 11c.OVA + αPD-L1 significantly greater than 11c.OVA + isotype and 11c.OVA + αPD-1 at day 3 (p<0.05), ** 11c.OVA + αPD-L1 significantly greater than 11c.OVA + isotype and 11c.OVA + αPD-1 at day 7 (p<0.01), (C, D) data pooled from 2 individual experiments of 2 mice per group with values for individual mice shown.

After adoptive transfer to 11c.OVA mice, OT-I Tcm-phenotype cells typically undergo a phase of clonal expansion prior to Bim-dependent contraction and inactivation [11]. Significantly, when antigen is presented by steady-state DC, substantial effector function is elicited prior to ‘tolerisation’ [12], consistent with demonstrations of transient effector function during tolerogenic antigen presentation in other model systems [34–36]. Administration of αPD-L1 substantially increased expansion of transferred OT-I Tcm-phenotype cells and delayed onset of contraction profoundly increasing of OT-I accumulation in 11c.OVA recipients (Fig. 2A). Blockade of PD-1, on the other hand, had a more modest effect on Tcm-phenotype cells (Fig. 2A) which differed to the outcomes observed for naive OT-I T cells. Accumulation of IFN-γ-producing OT-I was substantially increased by PD-L1 blockade and prolonged the persistence of this population exhibiting effector cytokine production compared to isotype-treated controls (Fig. 2B). As observed for OT-I Tcm-phenotype cells expansion, administration of αPD-1 had a modest effect relative to αPD-L1 treatment (Fig. 2B). As CTL activity may be regulated differently from proliferation and is important for adoptive immunotherapy we tested in vivo CTL activity. Despite the disparate effects of PD-1 or PD-L1 blockade on expansion and IFN-γ production, specific killing of OVA_257–264_-pulsed targets was significantly increased in 11c.OVA recipients by administration of either αPD-1 or αPD-L1, but αPD-L1 had a significantly greater effect 7 days after transfer (Fig. 2C). Additionally, administration of αPD-1 or αPD-L1 promoted CTL activity in 11c.OVA recipients compared to isotype controls for at least 2 weeks after OT-I Tcm-phenotype cell transfer (Fig. 2D). These data are consistent with PD-1/PD-L1 blockade creating a greater initial burst of effector cells and effector activity. To ensure that the disparate effects between blocking antibodies were not due to an ineffective administration regime for αPD-1, we titrated the amount of mAb administered and tested efficacy. At the dose used, increased OT-I accumulation was equivalent to that of an αPD-1 dose 2.5-fold higher (S2 Fig.)

### Blockade of PD-1/PD-L1 partially reverses inactivation of adoptively transferred OT-I Tcm-phenotype cells in 11c.OVA mice

**Fig 3:**
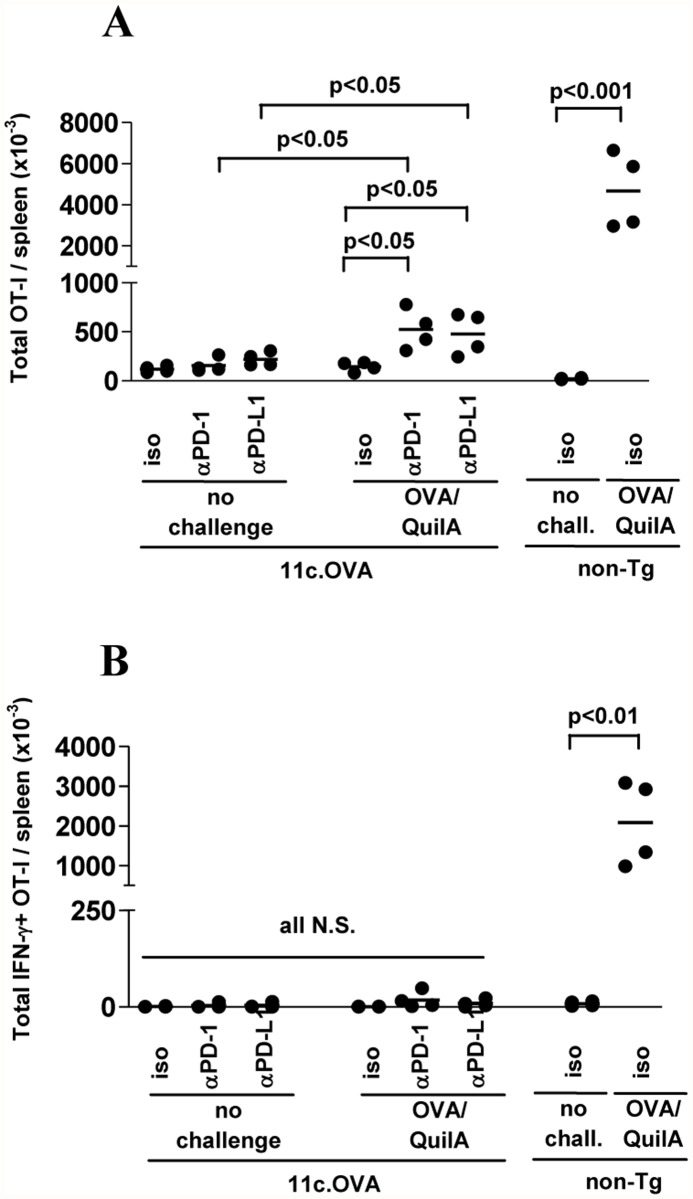
Blockade of PD-1/PD-L1 partially reverses non-responsiveness of tolerised memory CD8^+^ T cells. A, B) In vitro generated memory CD45.1^+^ OT-I T cells were transferred to 11c.OVA or C57BL/6 mice. Twenty eight days later mice were treated with αPD-1, αPD-L1 or isotype control mAb every subsequent three days. Nine days after commencement of mAb treatment some mice were challenged with OVA/QuilA immunization and a further five days later mice were euthanized and tissues collected for analysis using a flow-cytometric counting assay and intracellular cytokine staining. Data comprise: (A) four mice pooled from 4 experiments with n = 1 for each treatment set. Values for individual mice shown.

To determine whether blockade of PD-1/PD-L1 could restore function of OT-I Tcm-phenotype cells once they had become inactivated by tolerogenic DC-presented antigen in 11c.OVA mice, OT-I Tcm-phenotype cells were transferred to 11c.OVA or non-Tg recipients and 28 days later αPD-1 or αPD-L1 administered. After 9 days mAb treatment, mice were challenged with OVA/QuilA and 5 days later mice analysed. OVA/QuilA challenge led to a dramatic expansion (>200-fold) of OT-I T cells in non-Tg recipients (Fig. 3A, right) which was unaltered by αPD-1 or αPD-L1 mAb (not shown). In 11c.OVA recipients, administration ofαPD-1 or αPD-L1 mAb in the absence of OVA/QuilA challenge did not significantly alter the number of OT-I T cells present in spleen, indicating that even in the presence of DC-presented cognate antigen, blockade of PD-L1 or PD-1 did not result in expansion of residual inactivated OT-I T cells. OVA/QuilA challenge did not induce expansion of OT-I T cells in isotype-treated 11c.OVA recipients (Fig. 3A) indicating complete inactivation of OT-I Tcm-phenotype cells. However, αPD-1 or αPD-L1 treatment facilitated expansion of OT-I (3.6 and 3.3-fold) after OVA/QuilA challenge (Fig. 3A). While OVA/QuilA challenge substantially increased (approx. 250-fold) the total number of splenic IFN-γ-producing OT-I T cells in non-Tg recipients (Fig. 3B), OVA/QuilA challenge with or without blockade failed to increase IFN-γ-producing OT-I T cells in 11c.OVA recipients.

Together, the data indicate that administration of αPD-1 and, more profoundly, αPD-L1 promotes expansion and effector differentiation of OT-I Tcm-phenotype cells and prolongs the transient phase of effector function exerted by Tcm-phenotype cells encountering DC tolerogenically presenting antigen in the 11c.OVA environment but fails to effectively reverse the unresponsiveness of transferred Tcm-phenotype cells once inactivated.

### Impairment of Tcm-phenotype cells inactivation by PD-1 and PD-L1 blockade promotes effectiveness of adoptive immunotherapy

**Fig 4:**
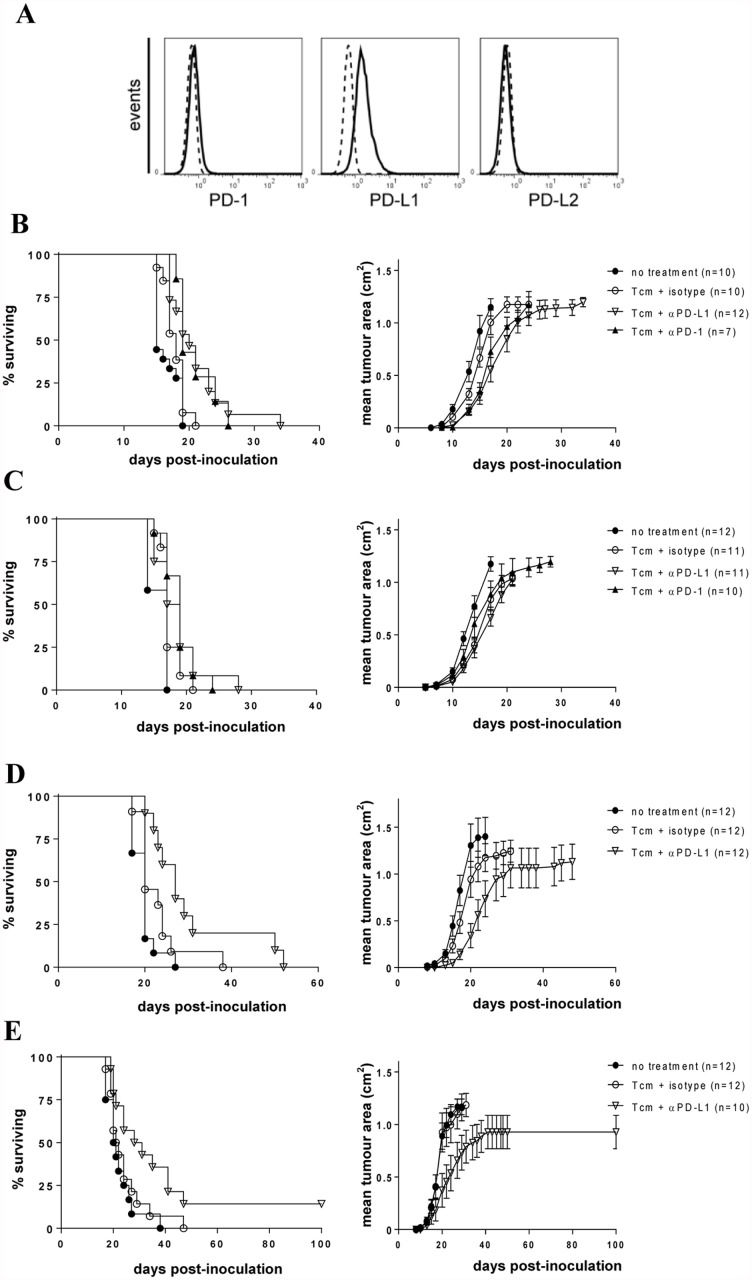
Anti-PD-1 and anti-PD-L1 synergises with Tcm-phenotype cell transfer to a tolerogenic environment. A) B16.mOVA cells were analysed for expression of PD-1 and PD-1 ligands. Histogram plots show specific antibody (solid line) and isotype control (dashed line) stained cells. Data are from a single analysis representative of 3 separate analyses. B) B16.mOVA cells (10^5^) were injected s.c. to 11c.OVA mice. Mice were left untreated (●) or 3 days later OT-I Tcm-phenotype cells (5 x 10^6^) transferred i.v. OT-I recipients were injected on the day of OT-I transfer and every subsequent 3 days with isotype control (◯), αPD-1 (▲) or αPD-L1 (▽) mAb. C-E) B16.mOVA cells (10^5^, C; 0.5 x 10^5^, D; 0.25 x 10^5^, E) were injected s.c. to C57BL/6 (C) or 11c.OVA mice (D, E). Mice were left untreated (●) or 3 days later OT-I Tcm-phenotype cells (5 x 10^6^) transferred i.v. OT-I recipients were injected on the day of OT-I transfer and every subsequent 3 days with isotype control (◯), αPD-1 (▲) or αPD-L1 (▽) mAb. Data show survival curves (left) or cumulative mean tumour area ± SEM (right).

To this point, interrupting PD-1/PD-L1 interactions profoundly impaired inactivation of Tcm-phenotype cells and promoted, albeit transiently, effector function of Tcm-phenotype cells transferred into a tolerogenic environment. To investigate whether adoptive immunotherapy using Tcm-phenotype cells would be enhanced by PD-1/PD-L1 blockade we explored a system employing stable transfectants of B16F0 (B16.mOVA) that expressed an identical form of OVA to that expressed by DC in 11c.OVA mice. B16.mOVA cells expressed modest levels of PD-L1 and no detectable PD-L2 (Fig. 4A).

Next we tested whether adoptive immunotherapy would exert immune pressure to impair tumour development in the tolerogenic 11c.OVA environment and whether PD-1/PD-L1 blockade might enhance this using a dose of tumour cells modelling a high pre-existing tumour burden. Tumours developed rapidly and consistently in 11c.OVA mice after inoculation of 10^5^ B16.mOVA cells (MST 15 days, Fig. 4B). Transfer of OT-I Tcm-phenotype cells together with isotype mAb modestly slowed tumour growth (MST 15 vs 18 days; p = 0.0399). αPD-1 or αPD-L1 together with OT-I Tcm-phenotype cells significantly slowed tumour growth compared to no treatment or OT-I Tcm-phenotype cell transfer and isotype control mAb (Fig. 4B) (MST 19 or 20 days; p = 0.0178 or 0.0230, respectively) and average tumour size was reduced (0.55 vs 1.01 cm^2^; p<0.05) at day 17 when 90% of untreated tumours had reached the maximum ethically-permitted size indicating a positive therapeutic effect that slows tumour development.

To distinguish whether PD-1 blockade was impairing tumour growth by i) inhibiting OVA-expressing DC-induced tolerance and promoting OT-I Tcm-phenotype cells effector function or ii) non-specifically promoting tumour clearance by endogenous non-OVA-specific T cells or other immune mechanisms, we compared B16.mOVA growth in non-Tg mice. Tumour development was identical in C57BL/6 and 11c.OVA mice (MST 17 vs 15 days; p>0.05, Fig. 4C vs 4B) suggesting that, in the absence of OT-I Tcm-phenotype cell transfer, endogenous OVA-specific immunity which would differ between OVA-tolerant 11c.OVA and non-Tg mice, or other immune mechanisms was playing little role in tumour clearance. OT-I Tcm-phenotype cells modestly but significantly slowed tumour growth in non-Tg mice (p = 0.0417). However, unlike in 11c.OVA mice, co-administration of αPD-1 or αPD-L1 together with OT-I Tcm-phenotype cells did not further limit tumour development (Fig. 4C). This indicates PD-1/PD-L1 blockade impairs the tolerogenicity of the 11c.OVA environment, promoting therapeutic effector function in transferred OT-I Tcm-phenotype cells in this environment. Treatment with αPD-1 or αPD-L1 did not alter tumour growth in non-Tg recipients without adoptively transferred OT-I Tcm-phenotype cells (S3 Fig.).

We next pursued studies using a reduced tumour burden to determine whether complete inhibition of tumour development could be achieved. As administration of αPD-1 and αPD-L1 gave statistically similar outcomes in tumour development studies to this point, we tested only αPD-L1. When a 2-fold reduced number of tumour cells was used, tumour development was slowed somewhat for untreated recipients (MST 15 vs 20 days) but tumour development still proceeded indicating the aggressiveness of the B16.mOVA tumour cells. Transfer of OT-I Tcm-phenotype cells did not change median survival time, however, tumour development was substantially slowed (MST 27 vs 20 days; p = 0.036) and average tumour size reduced (0.449 vs 1.08 cm^2^; p<0.01) when PD-L1 blockade was added to Tcm-phenotype cell transfer (Fig. 4D) and in some cases this more than doubled survival time. If a further 2-fold reduction was made, tumour development in untreated mice was affected only marginally (MST 20 vs 21 days) and adoptive transfer of Tcm-phenotype cells with isotype control mAb, again, did not significantly limit tumour development (Fig. 4E). Combining PD-L1 blockade, even for only 15 days, with OT-I Tcm-phenotype cells transfer here, however, profoundly slowed tumour development (MST 29.5 vs 21.5 days; p = 0.035), reduced average tumour size at day 27 (0.659 vs 1.097 cm^2^; p<0.05) and led to complete tumour inhibition in 20% of mice (Fig. 4E). Together these data show that in a ‘tolerogenic’ environment the effectiveness of adoptive Tcm-phenotype cell immunotherapy can be promoted by interruption of the PD-1/PD-L1 axis.

### Blockade of PD-L1 promotes prolonged accumulation of functional tumour-specific CD8^+^ T cells at the tumour site

**Fig 5:**
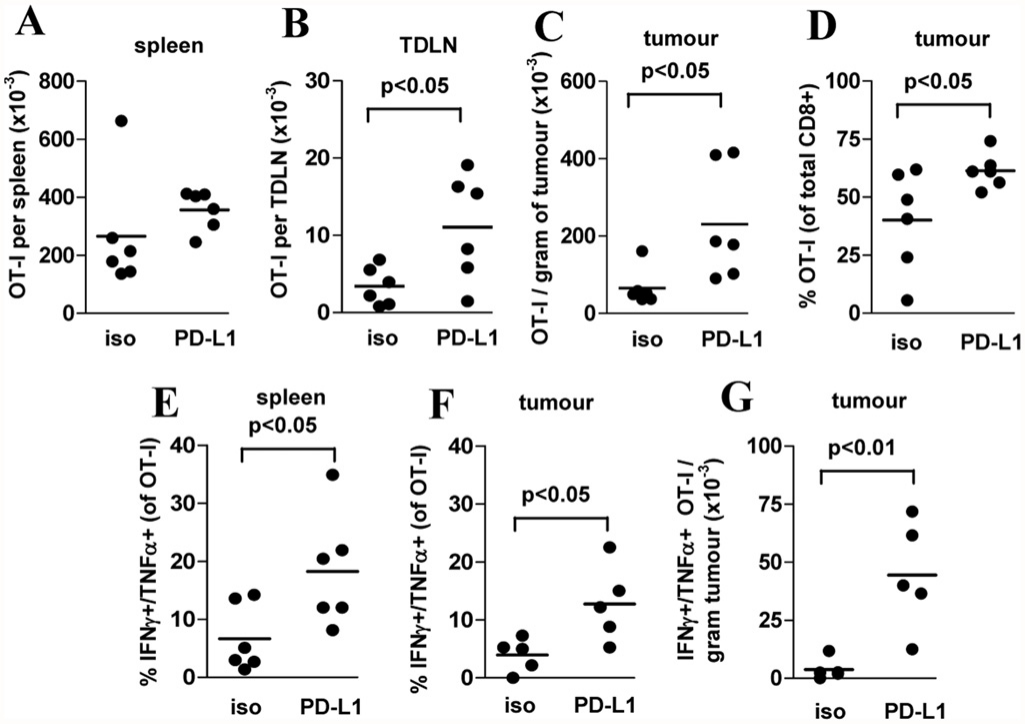
Anti-PD-L1 treatment increases tumour-specific CD8^+^ T cell infiltration and promotes effector function within tumours. A-G) B16.mOVA cells (10^5^) were injected s.c. to 11c.OVA mice and 3 days later OT-I Tcm-phenotype cells (5 x 10^6^) transferred i.v. OT-I recipients were injected on the day of OT-I transfer and every subsequent 3 days with isotype control or αPD-L1 mAb as indicated. Twelve days after OT-I Tcm-phenotype cell transfer, spleen (A, E), tumour draining LN (TDLN) (B), and tumour (C, D, F, G) were harvested and OT-I (CD45.1^+^/CD8^+^/Vα2^+^) number and cytokine production determined using a flow-cytometric counting assay (A, B, C, D) and intracellular cytokine staining (E, F) or a combination of both. Data represent individual mice pooled from 2 experiments of 2–3 mice per group.

To determine mechanisms of tumour inhibition by PD-1/PD-L1 blockade, the number and function of OT-I T cells 12 days after Tcm-phenotype cell transfer was determined. While the number of OT-I T cells in spleen did not differ between isotype and αPD-L1-treated mice (Fig. 5A) OT-I number in the tumour-draining (inguinal) LN total OT-I number was increased overall in αPD-L1-treated mice (Fig. 5B). Similarly, the density of OT-I T cells within tumours was also significantly increased (Fig. 5C) as was the proportion of OT-I T cells within tumour-infiltrating CD8^+^ T cells (Fig. 5D). The number of tumour-infiltrating CD4^+^ T cells or myeloid cells did not differ between isotype and αPD-L1-treated mice (not shown). αPD-L1 treatment also increased the proportion of OT-I T cells in spleens (Fig. 5E) and the tumour site (Fig. 5F) co-producing IFN-γ and TNF-α in response to *in vitro* OVA_257–264_ stimulation and also increased the absolute number of these cells in tumours (Fig. 5G) at a time when these populations are normally waning in peripheral lymphoid sites of 11c.OVA mice (e.g. Fig. 2).

**Fig 6:**
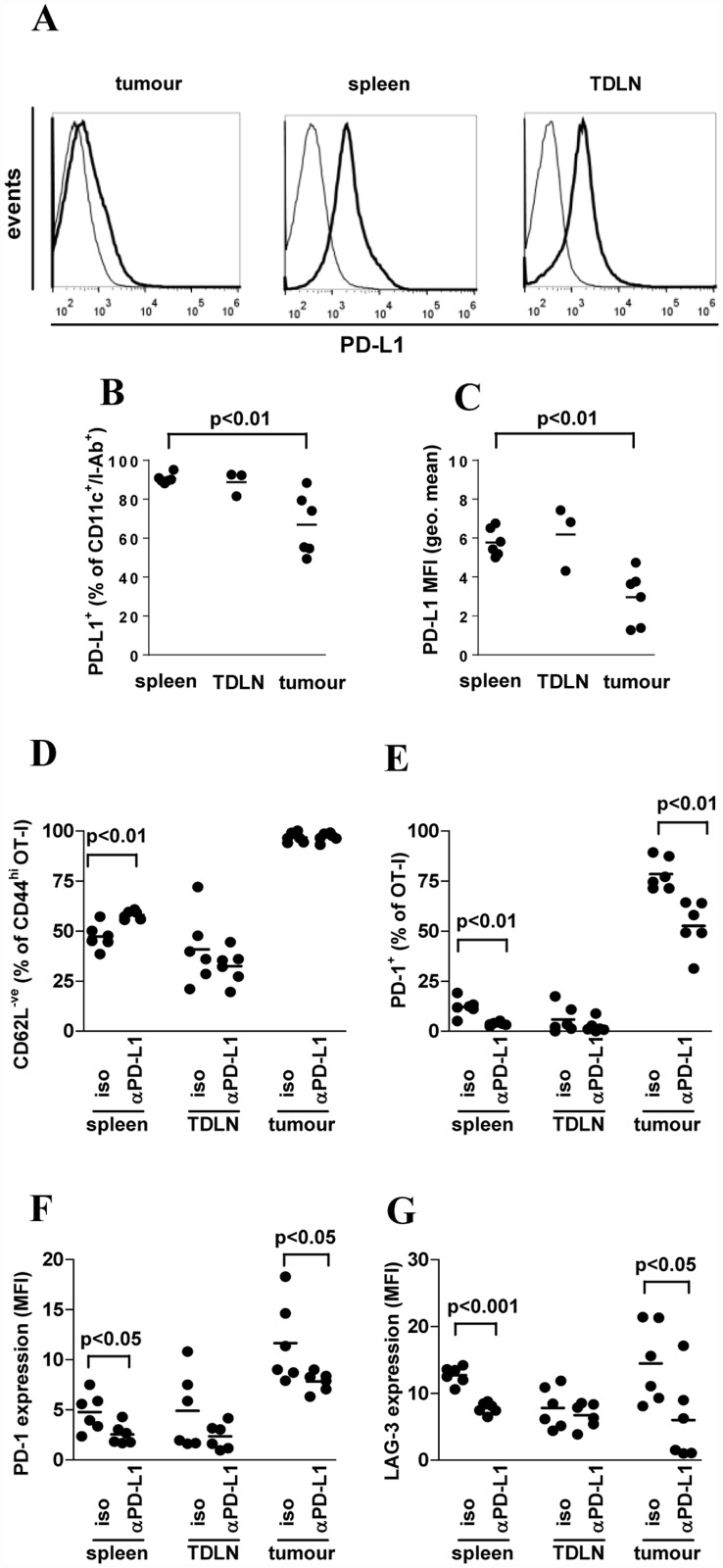
Anti-PD-L1 treatment reduces exhaustion marker expression on tumour-specific CD8^+^ T cells. A-G) B16.mOVA cells (10^5^) were injected s.c. to 11c.OVA mice and 3 days later OT-I Tcm-phenotype cells (5 x 10^6^) transferred i.v. OT-I recipients were injected on the day of OT-I transfer and every subsequent 3 days with isotype control or αPD-L1 mAb as indicated. Twelve days after OT-I Tcm-phenotype cells transfer, spleen, tumour draining LN (TDLN), and tumour sites were harvested for flow-cytometric analysis. A) PD-L1 expression was determined in tumour, spleen and TDLN (A) gated on the total bulk population for isotype control mAb-injected (solid line) mice. Isotype-control staining of tumour is shown (dashed line). B, C) the proportion of DC expressing PD-L1 (B) and PD-L1 expression level on DC (C) was determined (gated on CD11c^+^I-A^b+^ DC). CD62L and CD44 (D), PD-1 (E, F) and LAG-3 (G) expression was determined on OT-I T cells in spleen, TDLN and tumour. Data are representative of 3 mice/group in 2 separate experiments (A) or individual mice pooled from 2 separate experiments of 2–3 mice per group (B-G) except TDLN in (B, C) which is 3 mice from a single experiment of the 2 performed.

To understand where PD-L1 blockade effects may be mediated, we compared PD-L1 expression in the tumour and lymphoid tissues. Little PD-L1 was expressed within the tumour environment (Fig. 6A, left panel) where high expression of PD-L1 appeared restricted to infiltrating CD45^+^ immune cells. Low expression of PD-L1 by tumour cells or DC was not due to sensitivity of PD-L1 to the digest procedure (S4 Fig.). In contrast, in spleen and TDLN a large proportion of cells expressed substantial PD-L1 (Fig. 6A middle, right panels) and PD-L1 was expressed on the majority of DC in spleen, TDLN and tumour but the proportion and overall level of expression was lower on DC in tumour than in spleen (Fig. 6B, C). In spleen and TDLN close to half of all OT-I T cells displayed a CD62L^-ve^ effector phenotype (Fig. 6D) whereas in the tumour site, virtually all cells exhibited this phenotype. This was not altered in the tumour site by blockade of PD-L1, but in spleen, αPD-L1 treatment led to a small but consistent increase in the proportion of CD62L^-ve^ effector phenotype OT-I (Fig. 6D). In spleen and TDLN, PD-1 was expressed at low levels on a small proportion of OT-I T cells (Fig. 6E, F). In the tumour site, PD-1 was expressed on a high proportion of OT-I T cells and at much higher levels than in lymphoid tissues. Blockade of PD-L1 reduced PD-1 expression on OT-I T cells in spleen but not TDLN (Fig. 6E, F) and in tumour PD-L1 reduced both the proportion of OT-I T cells expressing PD-1 and expression level (Fig. 6E, F). Although the proportion of OT-I cells expressing LAG-3 did not differ (not shown), LAG-3 expression was reduced on OT-I T cells in spleen and tumour of αPD-L1-treated mice relative to isotype-treated controls (Fig. 6G), consistent with a reduction in the ‘exhausted’ phenotype of OT-I T cells.

## Discussion

Adoptive immunotherapy is a promising therapeutic approach to clear and prevent recurrence of cancers, but is limited by poor persistence and loss of function of transferred T cells [6]. Here we show that blockade of the PD-1/PD-L1 axis limits inactivation of CD8^+^ Tcm-phenotype cells transferred into a strongly tolerogenic environment and promotes antigen-specific inhibition of tumour development. This suggests that therapeutic blockade of the PD-1/PD-L1 inhibitory pathway may be an effective addition to current adoptive immunotherapeutic approaches using CTL or CD8^+^ Tcm-phenotype cells.

PD-1 signalling determines the early fate outcomes of T-cell activation where it can promote development of effector function [18]. This is strongly evident where the absence of PD-1 impairs, and together with CTLA-4 deficiency, prevents induction of tolerance in naive CD8^+^ T cells resulting from transient presentation of cognate antigen by steady-state DC [25]. Our findings show PD-1/PD-L1 also functions similarly in naive CD8^+^ T cells during chronic tolerogenic antigen presentation even though tolerance is not ultimately prevented. While the influence of PD-1 signalling on naive T cells is well established, the influence on memory CD8^+^ T cells, such as those used clinically for adoptive immunotherapy, remains unexplored. During tolerogenic antigen-presentation to CD8^+^ memory T cells we find PD-1 exerts a similar influence as reported for naive CD8^+^ T cells [25]. Blockade of PD-1/PD-L1 promoted expansion of CD8^+^ T cells and this was more profound with αPD-L1 mAb administration. This contrasted with naive CD8^+^ T cells where the effects of xPD-1 and αPD-L1 were similar. The mechanisms by which blockade of PD-L1 may provide more substantial effects only for Tcm-phenotype cells is unclear, but might reflect interaction of PD-L1 with alternative binding partners to PD-1 such as CD80 [37] or potentially yet-to-be-defined interactions [38]. Interactions between CD80 and PD-L1 can bi-directionally impart CD80-mediated negative signals to the interacting T cells [37,39]. Such interactions can contribute to tolerance induction [39] and PD-L1 expressed on T cells limits the pathogenicity of adoptively transferred effector T cells, possibly through bystander effects within the transferred T cell population [40]. It is feasible that blockade of PD-L1 could inhibit these effects to promote effector function. Consistent with this, PD-L1 was expressed on OT-I Tcm-phenotype cells at the time of transfer (S1 Fig.) and the mAb used here (10F9G2) interrupts binding of PD-L1 to both PD-1 and CD80 [41]. Additionally, ligation of PD-L1 and PD-L2 on DC inhibits DC function [42] and interruption of this could promote DC function and T-cell activation independently of PD-1 blockade. Significantly, however, the greater effect of αPD-L1 on promoting effector function in Tcm-phenotype cells is transient, is reflected mostly in proliferation (Fig. 2A) rather than CTL activity (Fig 2C, D), and this may explain why there is only a subtle and minimally apparent difference between the effects of the 2 mAb on inhibition of tumour development (Fig. 4B), as this possibly reflects the CTL-dependence of tumour inhibition.

In contrast to the strong effect on CD8^+^ T cells prior to tolerance induction (Fig. 1A- naive, Fig. 2- Tcm-phenotype cells), blockade of PD-1/PD-L1 only weakly reversed the inactivated state of ‘tolerant’ OT-I T cells arising after steady-state DC activation of naive CD8^+^ T cells or CD8^+^ Tcm-phenotype cells and this was limited to proliferative capacity. This differs from observations in chronic viral infection where blockade of PD-1 / PD-L1 can promote substantial, but incomplete, restoration of function in ‘inactivated’ virus-specific CD8^+^ T cells. There are two likely explanations for such different observations. In chronic viral infections, PD-L1 is upregulated [43,44] or possibly by inflammation associated with infection or ongoing immune attack [45]. Under these conditions abundant PD-L1 can bind PD-1 on T-cells and blockade could effectively inhibit this. On the other hand, as reported here, where cognate antigen presentation is restricted to steady-state DC with low PD-1 ligand expression relative to virus-exposed DC [46], blockade will exert less effect. Multiple inhibitory pathways act together to limit T-cell function under conditions of chronic viral infection [33,47], tumour growth [13], and transient steady-state antigen presentation [48]. Our data imply that alternative pathways to PD-1 are most important for maintaining an inactivated state in T cells under non-inflammatory conditions of chronic antigen presentation where PD-1 ligands are not upregulated.

In adoptive immunotherapy, the outcome of T-cell transfer will be influenced by the presence of a tolerogenic environment in recipients, including tolerogenic antigen presentation by DC [49]. An intriguing aspect of tolerance induction, however, is a transient period during which naive CD4^+^ and CD8^+^ T cells exhibit effector function prior to complete inactivation [34–36]. We found that CD8^+^ memory/effector T cells similarly exert substantial effector functions prior to inactivation by steady-state DC [12]. We propose a scenario exists during adoptive immunotherapy where transferred tumour-specific T cells are activated by steady-state or functionally altered DC ultimately leading to ‘tolerance’. We used the 11c.OVA mouse to model this effect on tumour-specific CD8^+^ Tcm-phenotype cells. We show that PD-1/PD-L1 blockade substantially enhanced effector function elicited from adoptively tumour-specific Tcm-phenotype cells prior to their ultimate deletion/inactivation to significantly inhibit, and in some cases, prevent tumour outgrowth.

In mouse models, PD-1/PD-L1 blockade can promote anti-tumour responses without adoptive immunotherapy, particularly in combination with blockade of other inhibitory pathways, with or without immunisation [50,51]. Other studies have examined the effects of PD-1/PD-L1 blockade in adoptive T-cell transfer studies, however, most have used naive donor T-cell populations. After adoptive transfer of naive antigen-specific T-cells, blockade of the PD-1 co-inhibitory pathway appears most effective when combined with other manipulations such as vaccination, removal of Treg, blockade of other co-inhibitory pathways, lymphodepletion or combinations of these [19,22,52–57]. Here, however, we wished to model the approach of using in vitro-activated CD8^+^ T cells employed clinically for adoptive immunotherapy and in some engineered TCR approaches. Using this approach, we found that while blockade of PD-1/PD-L1 was not particularly effective for restoring function in CD8^+^ T cells once they had been inactivated, blockade promoted effector function prior to tolerisation. Some similarities of the effects of PD-1/PD-L1 blockade were apparent between previous studies where naive T cells were adoptively transferred and the current study. Most notably increased IFN-γ in the tumour site and accumulation of CD8^+^ T cells, the latter attributed to IFN-γ-induced chemokines present in the tumour site after blockade and lymphodepletion [56]. Two other studies using either CTL in an AML model [58] or CD44^hi^CD62L^+^ T cells bearing chimeric antigen receptors for Her-2 [59] show positive effects of PD-1 blockade. While the latter study, which used cells expressing a CD62L^+^ Tcm-like phenotype similar to the cells tested here, lymphodepletion and administration of IL-2 was also employed and the authors attribute reduction of MDSC as reported by others [51] at the tumour site with key role in the outcome. The results published to date do reflect the possibility that combination approaches, for example, combined PD-1 and CTLA4 blockade or combination with lymphodepletion or Treg ablation may be the most effective clinically and testing combination therapies with adoptively-transferred Tcm-phenotype cells would be useful.

In combination the data show that blockade of PD-L1 promotes adoptive immunotherapy, by increasing conversion of CD8^+^ Tcm-phenotype cells to CTL in the periphery, away from the tumour bed, and facilitating accumulation of these cells in the tumour site. Furthermore, by reducing DC-induced inactivation in lymphoid tissues, tumour-specific CD8^+^ T cells accumulating in the tumour site exhibit a ‘less exhausted’ phenotype after PD-L1 blockade. It is possible the ‘exhausted’ phenotype of tumour infiltrating T cells is reduced through local effects of PD-L1 blockade in the tumour site, but while our data argue against this, it cannot be completely excluded in these experiments. However, the data do highlight that in a tumour immunotherapy setting, rather than modulating only T-cell effector function in the tumour interface, blockade of the PD-1 pathway can mediate significant effects on T cell activation away from the tumour site. This is similar to findings in an autoimmune model where blockade of PD-L1 promoted development of CD8^+^ effector differentiation distal to the target tissue resulting in infiltration and tissue destruction [60]. This suggests an action for PD-1/PD-L1 blockade additional to the promotion of T-cell effector function during T-cell-tumour interactions at the tumour site that is typically considered the key role of PD-1/PD-L1 blockade [22,23] in an anti-tumour setting. Given that Tcm-like cells or Tscm are proposed to be most effective for immunotherapy and are being developed for this purpose, the findings are particularly pertinent for adoptive immunotherapy as Tcm traffic preferentially to secondary lymphoid tissues, where presumably in a cancer setting, they are antigen activated and then subsequently differentiate to effectors with anti-tumour activity.

Recent clinical trials with antibody blockade of PD-1 or PD-L1 show blockade of this pathway successfully promotes tumour clearance likely through reactivation of pre-existing tumour-specific T-cell immunity or reversal of T-cell inhibition in tumours sites [20,21]. This together with success of CTLA4 blockade [61] indicates that blockade of co-inhibitory pathways has a strong potential for tumour treatment. Here we show that blockade of co-inhibitory pathways will be useful not only for restoring effective T-cell function in tumour bearing patients but also for promoting the effectiveness of adoptive immunotherapy using CTL or Tcm-phenotype cells. In fact our data suggest that the latter may be more profound. Use of co-inhibition blockade could alleviate the need for more toxic and detrimental regimes such as immunodepletion as a means to maximise the effectiveness of adoptive immunotherapy. We demonstrate that even a short course of just 15 days of blocking antibody treatment was sufficient in some cases to completely prevent tumour outgrowth. The system explored here, where strong PD-1 ligation was principally restricted to T-cell / tolerogenic DC interactions, showed a strong therapeutic benefit of PD-1/PD-L1 blockade. It is likely, however, that additional therapeutic benefits would be apparent where PD-1 ligation additionally acts to limit T cell function within the tumour environment and this warrants further investigation.

## Supporting Information

S1 Fig In vitro generated Tcm-phenotype cells express elevated levels of PD-1. OT-I lymph node cells were cultured for 3 days in IL-2 + OVA_257–264_-supplemented cultures, washed and recultured for 2 days in IL-15 supplemented cultures as described in materials and Methods. A) Surface phenotype (CD44/CD62L on CD8^+^/propidium iodide^-ve^—gated cells) at time of transfer was determined. Percentages are shown in each quadrant. Data are representative of more than 15 analyses. B) Cells were analysed by flow cytometry each day for expression of PD-1, PD-L1, PD-L2 across the culture period as indicated. Data are representative of more than 3 analyses.
(PDF)

S2 Fig Dose of αPD-1 administered is not suboptimal. OT-I LN cells were cultured in IL-2/peptide, washed and then cultured in IL-15 as described in Materials and Methods, recovered and administered i.v. to 11c.OVA mice that had been injected with the indicated amount of mAb i.p. 3 days later spleens were harvested and the number of OT-I (CD45.1^+^/CD8^+^/Vα2^+^) T cells per spleen enumerated by flow cytometry. Pooled from 3 separate experiments (n = 8 PBS, n = 9 200μg, n = 8 500μg).
(PDF)

S3 Fig αPD-1 or αPD-L1 alone do not alter B16.mOVA growth in non-transgenic recipients in the absence of adoptively transferred OT-I Tcm. B16.mOVA cells (1x10^5^) were injected s.c. to C57BL/6 mice as indicated. Mice were left untreated (●) or injected every 3 days with isotype control (◯), αPD-1 (▲) or αPD-L1 (▽) mAb. Data show survival curves or mean tumour area (± SEM) derived from 8 mice per group (pooled from 2 experiments of 4 mice per group) or from 3 untreated controls (2 from 1 experiment and 1 from the other).
(PDF)

S4 Fig Digestion does not alter PD-L1 staining of B16.mOVA tumour or spleen DC. A) B16mOVA cells were cultured in medium alone (top) or containing collagenase/DNAse as described in Materials and Methods (bottom) and stained with αPD-L1. B) Spleen cells from non-Tg mice cells were cultured in medium alone (top) or containing collagenase/DNAse as described in Materials and Methods (bottom) and stained with αI-A^b^, αCD11c and αPD-L1. Cells were gated for DC (CD11c^+^, I-A^bhi^) and staining for PD-L1 shown. Data is from a single experiment of 2 performed with identical results.
(PDF)
