# Supplementary figures and images for: Blockade of PD-1/PD-L1 Promotes Adoptive T-Cell Immunotherapy in a Tolerogenic Environment
Source: PLoS One. 2015 Mar 5;10(3):e0119483. doi: 10.1371/journal.pone.0119483 (PMC4351071; doi:10.1371/journal.pone.0119483)

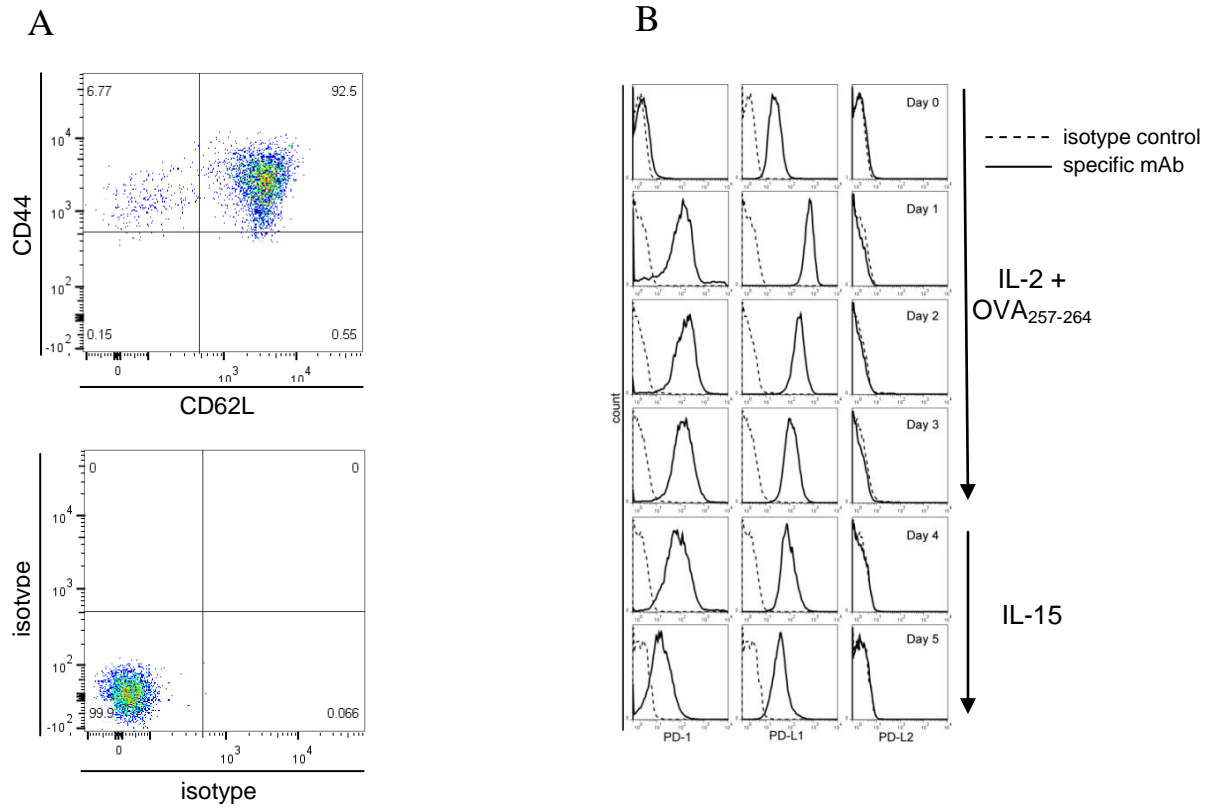

**S1 Fig.** In vitro generated Tcm-phenotype cells express elevated levels of PD-1.

Supplement: S1 Fig — OT-I lymph node cells were cultured for 3 days in IL-2 + OVA257–264-supplemented cultures, washed and recultured for 2 days in IL-15 supplemented cultures as described in materials and Methods. A) Surface phenotype (CD44/CD62L on CD8+/propidium iodide-ve—gated cells) at time of transfer was determined. Percentages are shown in each quadrant. Data are representative of more than 15 analyses. B) Cells were analysed by flow cytometry each day for expression of PD-1, PD-L1, PD-L2 across the culture period as indicated. Data are representative of more than 3 analyses. (PDF) [file pone.0119483.s001.pdf]

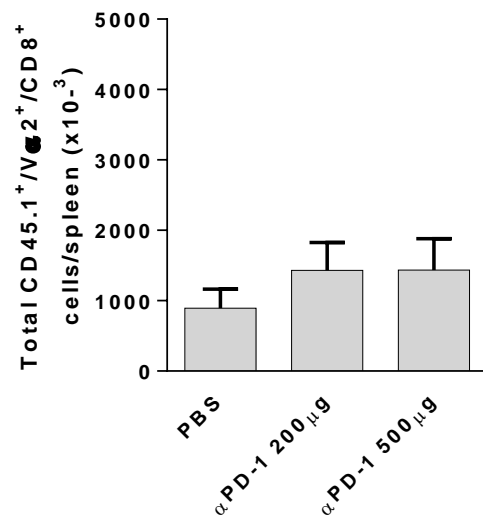

**S2 Fig.** Dose of αPD-1 administered is not suboptimal.

Supplement: S2 Fig — OT-I LN cells were cultured in IL-2/peptide, washed and then cultured in IL-15 as described in Materials and Methods, recovered and administered i.v. to 11c.OVA mice that had been injected with the indicated amount of mAb i.p. 3 days later spleens were harvested and the number of OT-I (CD45.1+/CD8+/Vα2+) T cells per spleen enumerated by flow cytometry. Pooled from 3 separate experiments (n = 8 PBS, n = 9 200μg, n = 8 500μg). (PDF) [file pone.0119483.s002.pdf]

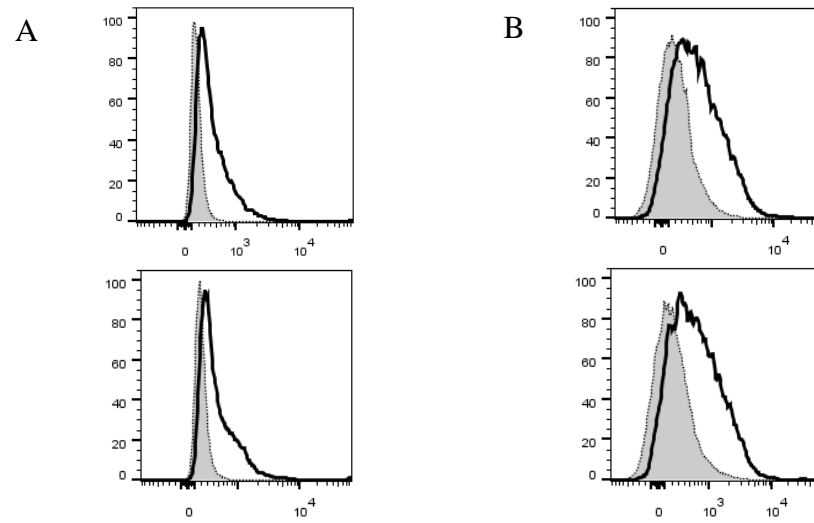

**S4 Fig. Digestion does not alter PD-L1 staining of B16.mOVA tumour or spleen DC.**

Supplement: S4 Fig — A) B16mOVA cells were cultured in medium alone (top) or containing collagenase/DNAse as described in Materials and Methods (bottom) and stained with αPD-L1. B) Spleen cells from non-Tg mice cells were cultured in medium alone (top) or containing collagenase/DNAse as described in Materials and Methods (bottom) and stained with αI-Ab, αCD11c and αPD-L1. Cells were gated for DC (CD11c+, I-Abhi) and staining for PD-L1 shown. Data is from a single experiment of 2 performed with identical results. (PDF) [file pone.0119483.s004.pdf]
