# Supplementary material for: Blockade of PD-1/PD-L1 Promotes Adoptive T-Cell Immunotherapy in a Tolerogenic Environment
Source: PLoS One. 2015 Mar 5;10(3):e0119483. doi: 10.1371/journal.pone.0119483 (PMC4351071; doi:10.1371/journal.pone.0119483)
Supplement: S3 Fig — B16.mOVA cells (1x10^5^) were injected s.c. to C57BL/6 mice as indicated. Mice were left untreated (●) or injected every 3 days with isotype control (◯), αPD-1 (▲) or αPD-L1 (▽) mAb. Data show survival curves or mean tumour area (± SEM) derived from 8 mice per group (pooled from 2 experiments of 4 mice per group) or from 3 untreated controls (2 from 1 experiment and 1 from the other). (PDF) [file pone.0119483.s003.pdf]

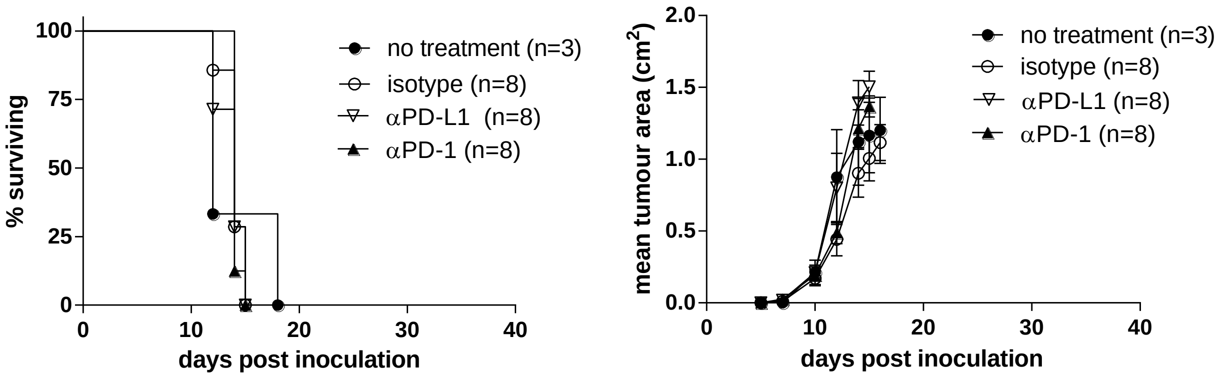

S3 Fig. αPD-1 or αPD-L1 alone do not alter B16.mOVA growth in non-transgenic recipients in the absence of adoptively transferred OT-I Tcm.
